# *lron-11* guides axons in the ventral nerve cord of *Caenorhabditis elegans*

**DOI:** 10.1371/journal.pone.0278258

**Published:** 2022-11-30

**Authors:** Nikolas Kokan, Skyla Witt, Saru Sandhu, Harald Hutter

**Affiliations:** Department of Biological Sciences, Simon Fraser University, Burnaby, BC, Canada; National Institutes of Health, UNITED STATES

## Abstract

For the nervous system to develop properly, neurons must connect in a precise way to form functional networks. This requires that outgrowing neuronal processes (axons) navigate to their target areas, where they establish proper synaptic connections. The molecular basis of this navigation process is not firmly understood. A candidate family containing putative receptors acting in various aspects of neuronal development including axon navigation are transmembrane proteins of the extracellular Leucine-Rich Repeat family (eLRRs). We systematically tested members of this family in *C*. *elegans* for a role in axon navigation in the ventral nerve cord (VNC). We found that *lron-11* mutants showed VNC navigation defects in several classes of neurons, including a pioneer neuron and various classes of interneurons and motoneurons. This suggests that while most members of the *lron*-family do not seem to have a role in axon navigation in the VNC, *lron-11* is likely to be a receptor required for correct navigation of axons in the VNC of *C*. *elegans*.

## Introduction

The nervous system originates during embryonic development when neural and glial precursor cells differentiate. To form functional neuronal circuits, neurons send out processes (axons and dendrites) to establish precise synaptic connections. Axons often navigate large distances to connect to their proper synaptic partners. Axons rely on receptors that detect guidance cues in their environment for accurate navigation. Several guidance cues and their receptors have been identified [1–3]. However, defects in genes encoding axon guidance cues or receptors are usually only partially penetrant, suggesting that we have only identified some of the genes controlling axon navigation.

**Fig 1:**
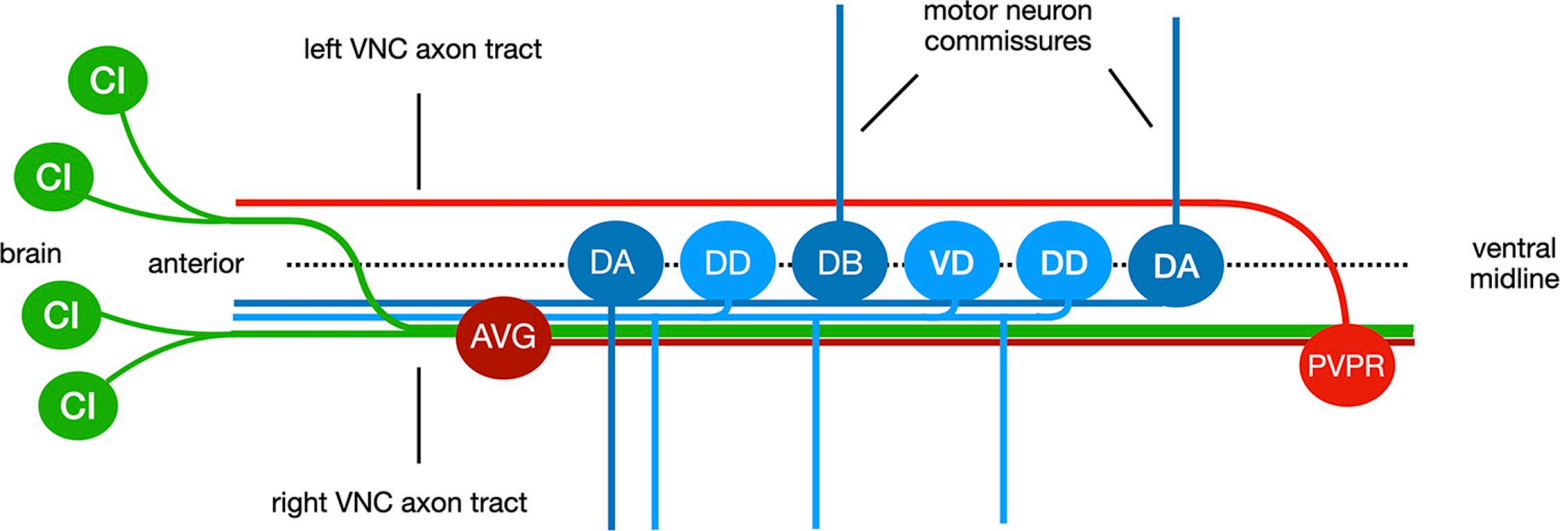
The ventral nerve cord. This schematic displays some of the neurons that send axons into the VNC. The AVG neuron cell body is located at the anterior end of the VNC. Its axon pioneers the right axon tract. The PVPR axon pioneers the left VNC axon tract, extending from the tail region in the anterior direction. Motoneuron cell bodies (DA, DB, VD and DD) are located between the two tracts of the VNC. They send neurites into the right tract, and commissures dorsally either on the left or the right side in an invariant pattern. Command interneurons (CI) extend axons from their cell bodies in the brain into the VNC. Axons entering from the left side cross into the right axon tract at the anterior end of the VNC.

The nematode *C*. *elegans* has a small nervous system of just 302 neurons, which have been grouped into 118 classes based on their position, morphology and synaptic connections [4]. The entire connectome has been mapped through serial sectioning electron microscopy [4]. This allows for a detailed analysis of neural circuits. Errors in nervous system development can be easily identified. Two major longitudinal axon bundles traverse the length of the animal, the dorsal nerve cord (DNC), and the ventral nerve cord (VNC) located on the dorsal and ventral side of the animal respectively. Of these, the VNC is the largest consisting of a small left axon tract and a much larger right axon tract (Fig 1). It is the *C*. *elegans* equivalent of the spinal cord of vertebrates.

Axons in a developing nervous system grow out sequentially. The first axons (pioneers) establish the initial axon tracts. Later outgrowing axons (followers) can–at least for part for their journey to their target area–‘follow’ the pioneer axons, simplifying their navigation problem. In *C*. *elegans* the VNC is pioneered by two neurons, AVG and PVPR [5]. AVG pioneers the right axon tract, and PVPR pioneers the left (Fig 1). Once the VNC axon tracts have been established by the pioneers, command interneuron axons (CI) enter the VNC from the brain, initially extending along both the left and right tracts. CI axons entering the VNC on the left side will soon cross into the right axon tract at the anterior end of the VNC (Fig 1). This results in an asymmetrical VNC with most axons extending along the right VNC axon tract. CI axons establish *en passant* synapses with motoneuron dendrites in the right VNC axon tract to control movement of the animal. Therefore, the correct positioning of an axon within the VNC is essential to successfully connect to synaptic partners [4, 6]. Cell bodies of various classes of motoneurons (e.g. DA, DB, DD, VD) are located between the two axon tracts, spread out along the ventral midline of the VNC (Fig 1). All motoneurons send their neurites into the right VNC axon tract. Motoneurons innervating dorsal muscles cells send commissures in an invariant pattern either up the right or the left side of the body to the DNC, where they connect to dorsal muscle cells. While a number of the guidance cues and receptors that guide these axons have been identified, defects in the corresponding mutants are only partially penetrant. This suggests that additional guidance cues and receptors remain unidentified.

**Fig 2:**
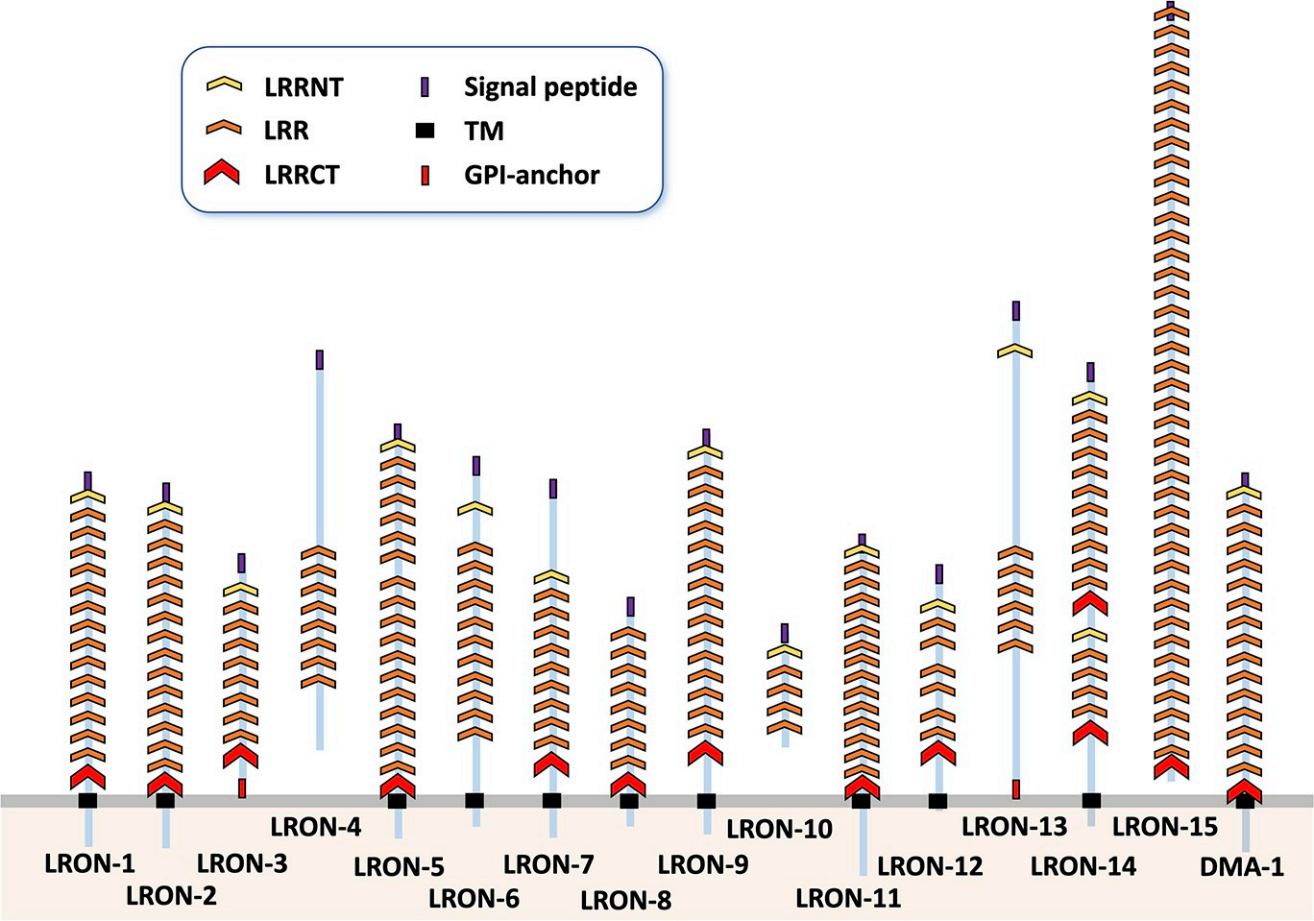
Domain organization of LRON-family members. Leucine-Rich Repeat (LRR) domains are depicted by orange chevrons, LRRNT caps (cysteine rich domains that often flank repeated LRR sequences) are yellow chevrons, and LRRCT caps are red chevrons. Transmembrane domains are in black, the signal peptides are purple, and GPI-anchors are red. The domain organization was determined by consulting SMART, Interpro and Phobius domain predictions [18–20]. LRR domains were also compared to the domains predicted by LRRscan [7].

Here we investigate whether the extracellular-Leucine Rich Repeat-Only (LRON) family in *C*. *elegans* contains novel axon guidance genes. The LRON family consists of 16 genes, which are characterized by only having Leucine-Rich Repeats (LRRs) in their extracellular domain (Fig 2). LRRs are a common protein domain mediating protein-protein interactions. Most LRON members are single pass transmembrane proteins, but LRON-3 and LRON-13 are GPI-anchored proteins and LRON-4 and LRON-10 are secreted proteins. Therefore, most LRON proteins are putative receptors. While these proteins share a general domain composition, due to sequence divergence during evolution most of the family members do not show much similarity at the sequence level, with each other or with extracellular LRR proteins in other species [7]. This prevents straightforward identification of a mammalian homolog for members of the LRON family (7). For example, automated algorithms [8–10] predict that LRON-11 is a homolog of the mammalian protein carboxypeptidase N subunit 2 (CPN2), which seems unlikely since LRON-11 is a transmembrane protein whereas CPN2 is secreted. Like most LRON proteins, a large percentage of the LRON-11 protein is made up of LRR repeats, so it has modest sequence homology to many proteins like CPN2 that also have many LRR domains. However, the LRON family, including LRON-11, does share a similar protein structure with other mammalian extracellular LRR families, particularly with the LRR TransMembrane neuronal (LRRTM) family, suggesting that a member of this family may be a homolog of LRON-11. The prevalence of characterized extracellular LRR proteins in signal transduction during nervous system development makes LRON proteins candidates for potential axon guidance genes [7, 11]. Furthermore, various proteins containing extracellular LRR domains are involved in axon guidance [12–14]. This includes receptors as well as secreted guidance cues such as SLT-1/Slit [15–17].

Most *lron* genes have not been studied in *C*. *elegans*, though RNAseq experiments provide evidence that many of them are expressed in neural or hypodermal cells when axons are extending [21]. The exception is *dma-1*, which plays an important role in dendrite guidance and branching in the PVD neuron [22, 23]. DMA-1 is present in PVD dendrites and forms an adhesion complex with SAX-7/L1CAM and MNR-1 which are expressed in the adjacent hypodermis [23, 24]. Since there is evidence that other members of the *lron* family are also expressed in neurons or hypodermal cells, we were interested in systematically testing *lron*-genes for a potential role in axon guidance [21, 22, 25].

We surveyed members of the LRON family for a role in axon guidance in *C*. *elegans* focussing on the VNC, the main longitudinal axon tract. We selected putative null alleles in *lron*-genes from the strain collection centre Caenorhabditis Genetics Center (CGC). For our initial analysis we used a pan-neuronal marker to detect major axonal defects. We also used single cell markers for VNC pioneer axons to identify defects specifically in early outgrowing axons. We found that *lron-11* mutants had the highest penetrance of axon guidance defects scored with the pan-neuronal marker. To identify the neurons that were affected, we used cell-type specific markers for various classes of interneuron and motoneuron axons in the VNC. *lron-11* mutants had significant defects in command interneurons as well as several classes of motoneurons. Motoneuron defects included navigation defects in the VNC as well as polarity defects in commissures extending from the VNC to the DNC. Our results demonstrate that *lron-11* functions in the guidance of different types of axons in the *C*. *elegans* VNC.

## Material and methods

### Strains and genotyping

Strains were grown and maintained at 20°C or 15°C, following standard procedures [26]. The following fluorescent marker strains were used to observe axons: *hdIs26[odr-2*::*CFP*, *sra-6*::*DsRed2] III*, *hdIs28[odr-2*::*CFP*, *sra-6*::*DsRed2]*, *hdIs29[odr-2*::*CFP*, *sra-6*::*DsRed2]*, *evIs111[rgef-1*::*GFP] V*, *oxIs12[unc-47*:*GFPNTX;lin-15(+)] X*, *rhIs4[glr-1*::*GFP*, *dpy-20(+)] III*, *zdIs13[tph-1*::*gfp]*, *hdIs25[unc-129*::*CFP*, *unc-47*::*DsRed2]*, *hdIs54[flp-1*::*GFP]*, *evIs82A[unc-129*::*GFP] II*.

The following mutations in *lron*-genes were analysed: *lron-1(gk5081); lron-3(ok2614); lron-3(gk5319); lron-4(gk5099); lron-5(gk959442); lron-6(gk736335); lron-7(gk5353); lron-8(gk5317); lron-10(gk5064); lron-11(gk5321); lron-11(ok2333); lron-12(gk187625); lron-13(gkDf31); lron-14(gk401715); lron-15(gk918201); dma-1(wy686)*. For details about the molecular nature of the mutations, see S1 Table. All alleles are putative null alleles.

PCR was used to identify homozygous mutants after crossing with marker strains. For deletion alleles, size differences between wildtype and mutant were used with primers flanking the deletion. In addition, a primer within the deletion was used to confirm absence of a wildtype allele. For point mutations, the PCR product was sequenced. Primers used for genotyping are in S2 Table. Primers used for sequencing point mutations are in S3 Table.

### Phenotyping

Axon guidance defects were assessed in adult worms. Animals were incubated in 10mM sodium azide for an hour to immobilize the animals prior to mounting on 4% agar pads. A Zeiss Axioscope microscope (Carl-Zeiss AG, Germany) with a 40X magnification objective was used to evaluate axonal defects. Confocal images were taken on a Zeiss Axioplan II microscope (Carl-Zeiss AG, Germany) connected to a Quorum WaveFX spinning disc system (Quorum Technologies, Canada). Stacks of confocal images with 0.2 to 0.5 μm distance between focal planes were recorded. Image acquisition and analysis was carried out by using Volocity software (Quorum Technologies, Canada). Images were assembled using Keynote (Apple Inc).

### Statistics

A χ2 test was used to determine if the mutant’s axon guidance defects were significantly different from the marker strain.

## Results

### VNC axon guidance defects in *lron*-mutants

**Fig 3:**
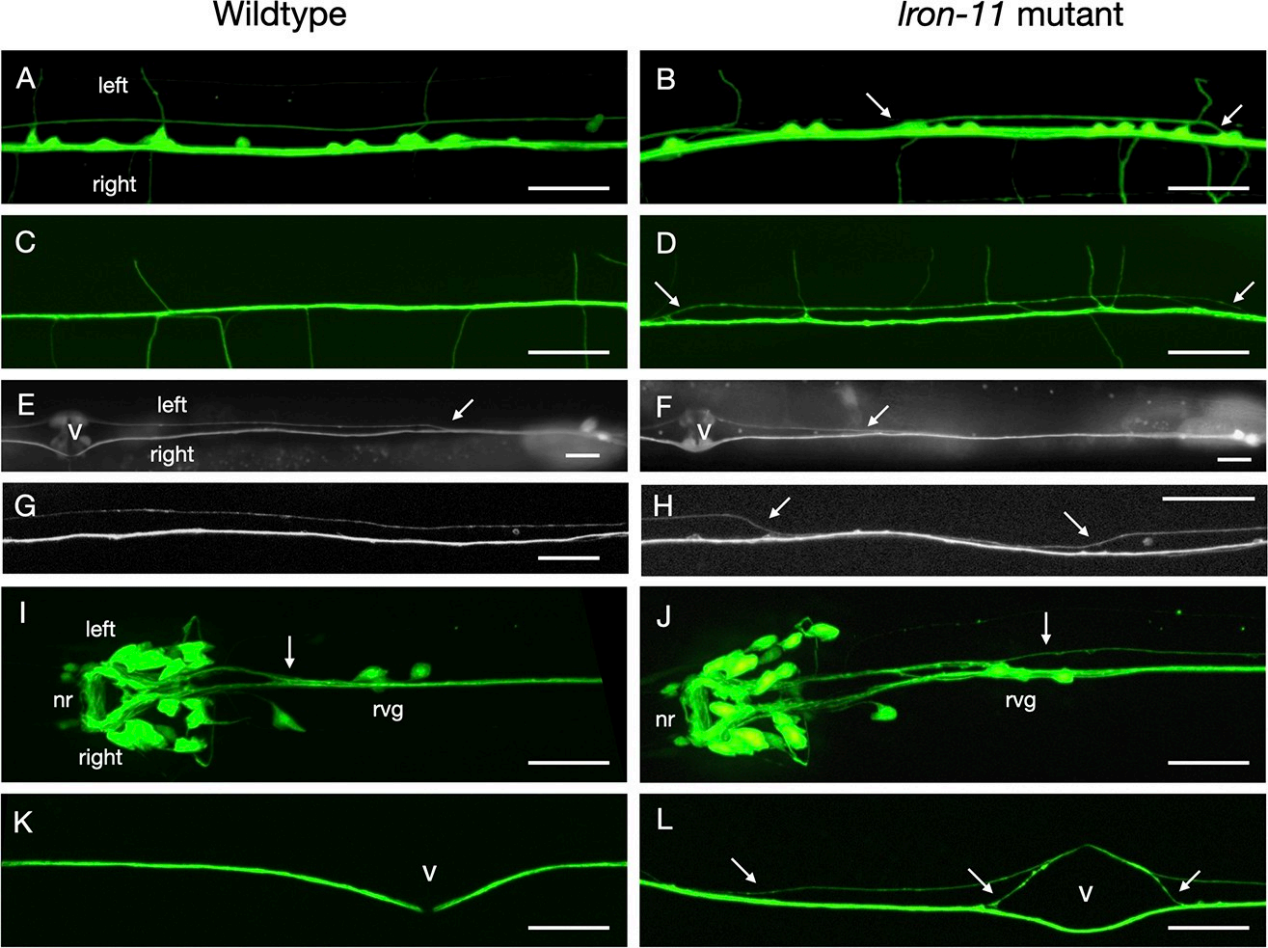
Pan-neuronal, pioneer and interneuron defects in *lron-11* mutants. Panels A, C, E, G, I, K show wildtype, panels B, D, F, H, J, L show *lron-11* mutants. The anterior side of the animals are towards the left of the image, the left side of the animals is towards the top of the image. Scale bars: 20μm. A, B: VNC visualized with the pan-neuronal marker *evIs111[rgef-1*::*GFP]*. A) In wildtype animals axons do not cross between left and right axon tracts. B) in *lron-11(ok2333)* mutant animals axons cross between the left and right axon tracts (arrows). C, D: DNC visualized with the pan-neuronal marker *evIs111[rgef-1*::*GFP]*. C) In wildtype animals axons are tightly fasciculated. D) in *lron-11(ok2333)* mutant animals axons are partially defasciculated (arrows). E-H: Pioneer axons (PVPR and AVG) visualized with *hdIs26[odr-2*::*CFP & sra-6*::*DsRed2]*. PVPR is the fainter axon in the left tract. AVG is the brighter axon below it, in the right tract. E) In wildtype animals PVPR crosses from the right tract into the left tract close to the posterior end of the VNC (arrow). F) In *lron-11(ok2333)* mutant animals the PVPR axons sometimes crosses into the left tract at a much more anterior position (arrow, ‘late separation defect’) close to the vulva (marked with a ‘v’). G) In wildtype animals pioneer axons stay in their axon tracts. H) In *lron-11(ok2333)* the PVPR axon sometimes crosses into the right axon tract and then back into the left axon tract (arrows). I-L: Command interneuron axons visualized with *rhIs4[glr-1*::*GFP]*. I) In wildtype animals CI axons entering the VNC from the nerve ring (nr) on the left side cross into the right axon tract anterior to the retrovescicular ganglion (rvg). J) In *lron-11*(*ok2333*) mutant animals, some CI axons are late crossing into the right tract (arrow). K) In wildtype animals all CI axons extend in the right axon tract (‘v’ marks the position of the vulva). L) In *lron-11*(*ok2333*) mutant animals, some CI axons cross into the left tract at various positions and sometime cross back (arrows).

To observe whether *lron* genes are required for proper axon guidance in the VNC, we used a pan-neuronal GFP marker to visualize all neurons and axons to survey for general VNC axon guidance defects in *lron* mutants. We found significant axon navigation defects in *lron-3(gk5319)*, *lron-8(gk5317)*, *lron-11(ok2333)* and *lron-14(gk401715)* mutants (Table 1). In these mutants, axons inappropriately crossed between right and left axon tracts (Fig 3B). We did not evaluate axonal defects in *lron-2* and *lron-9* mutants since null alleles in these genes are lethal. We used a second fluorescent marker to identify axonal defects in the VNC pioneer axons, PVPR and AVG. Most of the *lron* mutants did not show any significant pioneer axon navigation defects. However, *lron-5(gk959442)* and *lron-11(ok2333)* mutants had significant PVPR pioneer axon defects (Table 1 and Fig 3H). In addition, in *lron-11(ok2333)* mutants the AVG axon showed a low (4%) penetrance defects of crossing into the left VNC axon tract.

**Table 1:** VNC midline crossing defects of *lron*-mutants ^a^.

Genotype	% general VNC defects (n)	% PVPR defects (n)	% AVG defects (n)
Wild type	5 (787)	6–10 ^b^^,^^c^^,^^d^	0–1^b^^,^^c^^,^^d^
*lron-1*(*gk5081*)	4 (102)	4 ^b^ (100)	0 ^b^ (100)
*lron-3*(*gk5319*)	11* (102)	17 ^c^ (96)	2 ^c^ (96)
*lron-3*(*ok2614*)	10 (105)	9 ^b^ (100)	0 ^b^ (100)
*lron-4*(*gk5099*)	1 (96)	7 ^b^ (105)	0 ^b^ (105)
*lron-5*(*gk959442*)	8 (89)	23 ^d^ *** (94)	1 ^d^ (94)
*lron-5*(*gk5278*)	n.d.	3 (100)	0 (100)
*lron-6*(*gk736335*)	3 (99)	3 ^d^ (100)	1 ^d^ (100)
*lron-7*(*gk5353*)	8 (99)	9 ^c^ (100)	2 ^c^ (100)
*lron-8*(*gk5317*)	13** (100)	4 ^d^ (81)	0 ^d^ (81)
*lron-10*(*gk5064*)	6 (100)	11 ^d^ (102)	2 ^d^ (102)
*lron-11*(*ok2333*)	29*** (96)	15 ^b^ * (110)	4 ^b^ ** (110)
*lron-11*(*gk5321*)	28*** (96)	19 ^b^ *** (102)	1 ^b^ (102)
*lron-12* (*gk187625*)	10 (102)	4 ^d^ (93)	0 ^d^ (93)
*lron-13*(*gkDf31*)	7 (98)	7 ^d^ (108)	0 ^d^ (108)
*lron-14* (*gk401715*)	17*** (88)	15 ^c^ (101)	0 ^c^ (101)
*lron-14* (*gk5340*)	4 (100)	n.d.	n.d.
*lron-15* (*gk918201*)	7 (102)	5 ^b^ (101)	0 ^b^ (101)
*dma-1*(*wy686*)	5 (101)	3 ^b^ (75)	0 ^b^ (75)

***p<0.001;

**p<0.01;

*p<0.05 in comparison to the corresponding marker strain (χ2 test).

Marker strains used: *evIs111* for general VNC defects, *hdIs26*, *hdIs28*, *hdIs29* for PVPR and AVG defects.

^a^ All alleles used in this study are putative null alleles based on the molecular nature of the allele (see S1 Table)

^b^ Defects in *hdIs26*: 6% (n = 294)

^c^ Defects in *hdIs28*: 10% (n = 163)

^d^ Defects in *hdIs29*: 7% (n = 159)

To confirm that the observed axonal defects are due to the mutations in the *lron* genes rather than background mutations, we tested additional alleles (Table 1). With the pan-neuronal marker we found no significant defects in *lron-3(ok2614)*, *lron-5(gk5278) and lron-14(gk5340)* mutants (Table 1), whereas the nature and penetrance of defects in *lron-11(gk5321)* mutants were similar to those we observed in *lron-11(ok2333)*. This suggests that axonal defects in *lron-11* mutants are caused by a defect in *lron-11’*s function, whereas defects in the other three *lron*-mutants are most likely caused by unidentified background mutations, which are an inevitable by-product of the mutagenesis used to create these alleles. With the pioneer marker, we evaluated defects in *lron-5(gk5278)* and *lron-11(gk5321)*. The only significant pioneer navigation defects we found were PVPR navigation defects in *lron-11(gk5321)* mutants, suggesting that the defects observed in the *lron-11* mutant strains are not due to background mutations but due to the mutations in *lron-11* itself.

### Interneuron VNC axon guidance defects in *lron-11* mutants

Since *lron-11* mutants had the most penetrant axonal defects with the pan-neuronal marker as well as defects in PVPR pioneer axon navigation, we focussed on *lron-11* for a more detailed analysis of axonal defects in the VNC. To determine if there is a preferred region along the anterior-posterior axis, where axons crossed into the left axon tract, we subdivided the pan-neuronal crossover defects into ‘anterior’ versus ‘posterior’ defects, using the vulva located at the midbody region as the demarcation point. We did not find a significant difference between ‘anterior’ and ‘posterior’ crossovers (Table 2), suggesting that *lron-11* affects crossovers in general and not in a specific region. We also evaluated the dorsal nerve cord (DNC) and found rare but significantly penetrant defasciculation defects in *lron-11(gk5321)* mutants (Table 2 and Fig 3D).

**Table 2:** Panneuronal and interneuron axon guidance defects in *lron-11* mutants.

	% defects		
Neuron class	Wildtype	*lron-11(ok2333)*	*lron-11(gk5321)*
**Panneuronal–VNC**	n = 787	n = 96	n = 96
No Defect	95	71***	72***
Anterior Crossover	2	15***	19***
Posterior Crossover	3	19***	13***
Late Separation	0	4***	2
**Panneuronal–DNC**	n = 245	n = 100	n = 98
Defasciculation	1	4	8 **
**PVPR**	n = 294	n = 110	n = 102
No Defect	94	85**	81***
Crossovers	6	10	13*
Late Separation	1	3	5*
**Command Interneurons**	n = 134	n = 97	
No Defect	98	82**	n.d.
Crossover	0	5**	n.d.
Late Crossover to Right Tract	2	11**	n.d.
**HSN and AVK**	n = 32–64	n = 104–115	
HSNL	91	78	n.d.
HSNR	95	90	n.d.
AVKL	100	94	n.d.
AVKR	100	92	n.d.

***p<0.001;

**p<0.01;

*p<0.05 (χ2 test). Markers used: *evIs111[rgef-1*::*GFP]* for DNC and VNC defects, *hdIs26[odr-2*::*CFP*, *sra-6*::*DsRed2]* for PVPR defects, *rhIs4[glr-1*::*GFP*, *dpy-20(+)]* for command interneuron defects, *zdIs13[tph-1*::*gfp]* for HSN defects and *hdIs54[flp-1*::*GFP]* for AVK defects. Individual animals can have more than one defect, e.g. “anterior crossover” and “posterior crossover”, so that numbers do not always add up to 100%.

The PVPR pioneer axon navigation defects in *lron-11* mutants can be subdivided into two categories. First a ‘late separation’ defect, where the PVPR axon stays in the right axon tract until at least halfway to the vulva before eventually crossing the midline and pioneering the left axon tract (Fig 3F), and secondly a crossover defect, where the axon crosses into right axon tract (Fig 3H). The majority of defects in both *lron-11* alleles were crossover defects (Table 2).

The major class of interneurons in the VNC are the ‘command interneurons’ (CI), which innervate the motoneurons that control movement of the animal. CI cell bodies are located in the head and their axons extend into the VNC from the brain. All CI axons entering the VNC from the left side of the brain cross into the right VNC axon tract at the anterior of the VNC (Fig 1) near the retrovesicular ganglion (rvg). We observed two types of CI axon navigation defects in *lron-11* mutants. A ‘late crossover’ defect, where CI axons entering the VNC from the brain crossover into the right tract posterior of the rvg (Table 2 and Fig 3J) and a crossover defect, where axons cross from the right to the left axon tract (Table 2 and Fig 3L). The ‘late crossover’ defects were similar between the two *lron-11* alleles (11% and 10%), whereas the crossover defects were more penetrant in *lron-11(gk5321)* (13% versus 5% in *lron-11(ok2333)*. Overall, interneuron defects in the stronger allele, *lron-11(gk5321)*, were moderately penetrant (23%). We also observed the AVK interneurons and the HSN motoneurons, however we did not find significant axonal defects in the VNC (Table 2). In a small number of animals (4%) one of the AVK axons leaves the nerve ring in the head prematurely and extends laterally (S1 Fig). However, these defects are not statistically significant.

### Motoneuron VNC axon guidance defects in *lron-11* mutants

**Fig 4:**
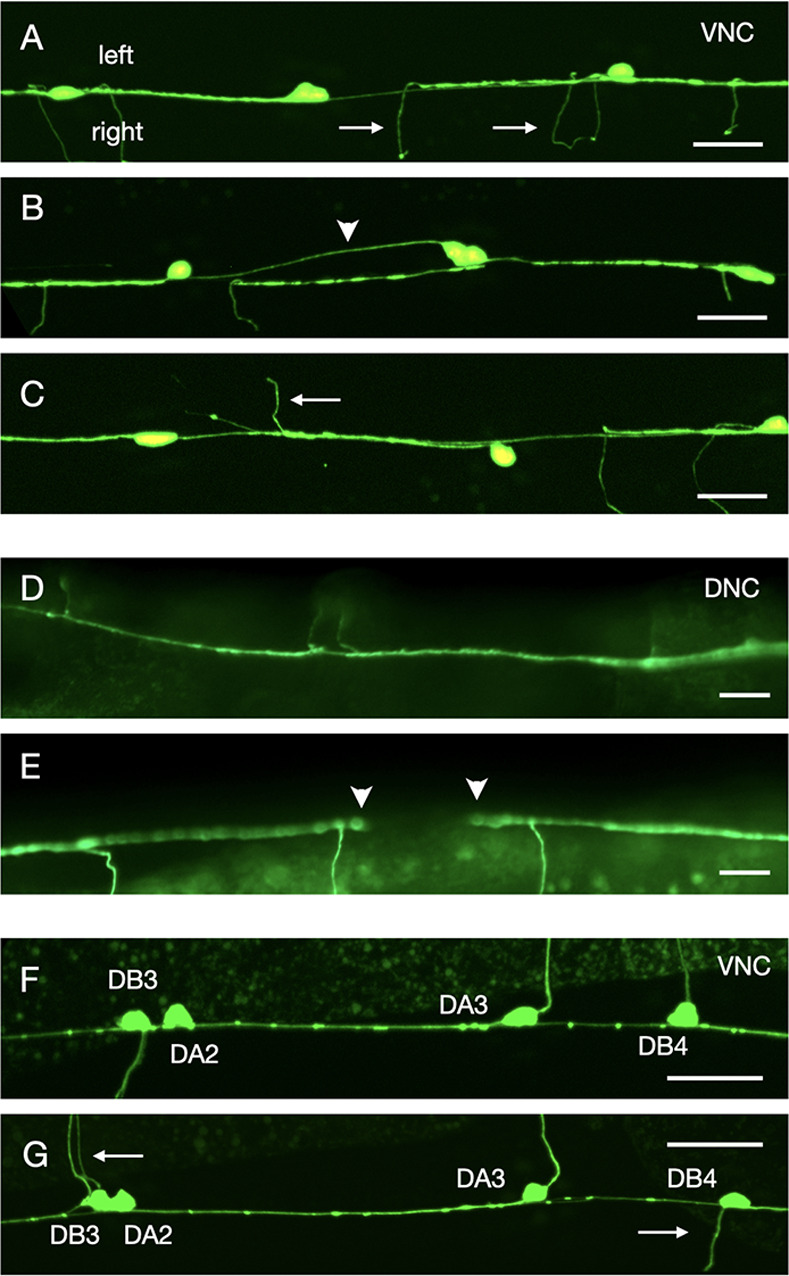
Motoneuron defects in *lron-11* mutants. Panels A, D and F show wildtype, panels B, C, E and G show *lron-11* mutants. The anterior side of the animals are towards the left of the image, the left side of the animals is towards the top of the image. Scale bars: 20μm. A-C: VNC visualized with the DD/VD motoneuron marker *oxIs12[unc-47*::*GFP)]*. A) In wildtype DD/VD neurites are in the right axon tract and commissures extend on the right side (arrows). B) In *lron-11(ok2333)* mutant animals some axons extend in the left axon tract (arrowhead). C) In *lron-11(ok2333)* mutant animals some commissures extend on the left side (arrow). D, E: DNC visualized with the DD/VD motoneuron marker *oxIs12[unc-47*::*GFP)]*. D) In wildtype animals motoneuron axons cover the entire DNC without gaps. E) Some *lron-11(ok2333)* mutant animals show gaps in the DNC (arrowheads). F-G: VNC visualized with the DA/DB motoneuron marker *evIs82A[unc-129*::*GFP]*. The images contain the region covering the neurons DB3, DA2, DA3 and DB4 in order, from left to right. F) In wildtype animals DB3 and DA2 send commissures to the right side, whereas DA3 and DB4 send their commissures to the left side. G) In this *lron-11*(*ok2333*) mutant animal, the DB3, DA2 and DB4 commissures extend on the wrong side (arrows).

Several classes of motoneurons extend neurites into the right VNC axon tract. We found that in 22% of *lron-11(ok2333)* mutants, DD/VD neurites extend into the left VNC axon tract (Table 3 and Fig 4B).

**Table 3:** Motoneuron axon guidance defects in *lron-11* mutants.

	% defects	
Neuron class	Wildtype	*lron-11(ok2333)*
**DA/DB commissure polarity defects**	n = 50	n = 101
No Defect	94	24 ^a^ ***
Single Commissure	4	34 ^a^ ***
Two or Three Commissures	2	35 ^a^ ***
Four or More Commissures	0	7
**DD/VD commissure polarity defects**	n = 58	n = 101
No Defect	66	45*
Single Commissure error	26	36
Two or Three Commissures errors	7	20
**DD/VD neurites in the left VNC**	n = 58	n = 101
No Defect	97	78**
Neurite(s) in the Left Tract	3	22**
**DD/VD gaps in the DNC**	n = 54	n = 97
No Defect	91	77*
One Gap in the DNC	7	18
Two or More Gaps in the DNC	2	5

*p<0.05 (χ2 test). Markers used: *oxIs12[unc-47*:*GFPNTX;lin-15*(+)] for DD/VD neurons, *evIs82A[unc-129*::*GFP]* for DA/DB neurons.

**Fig 5:**
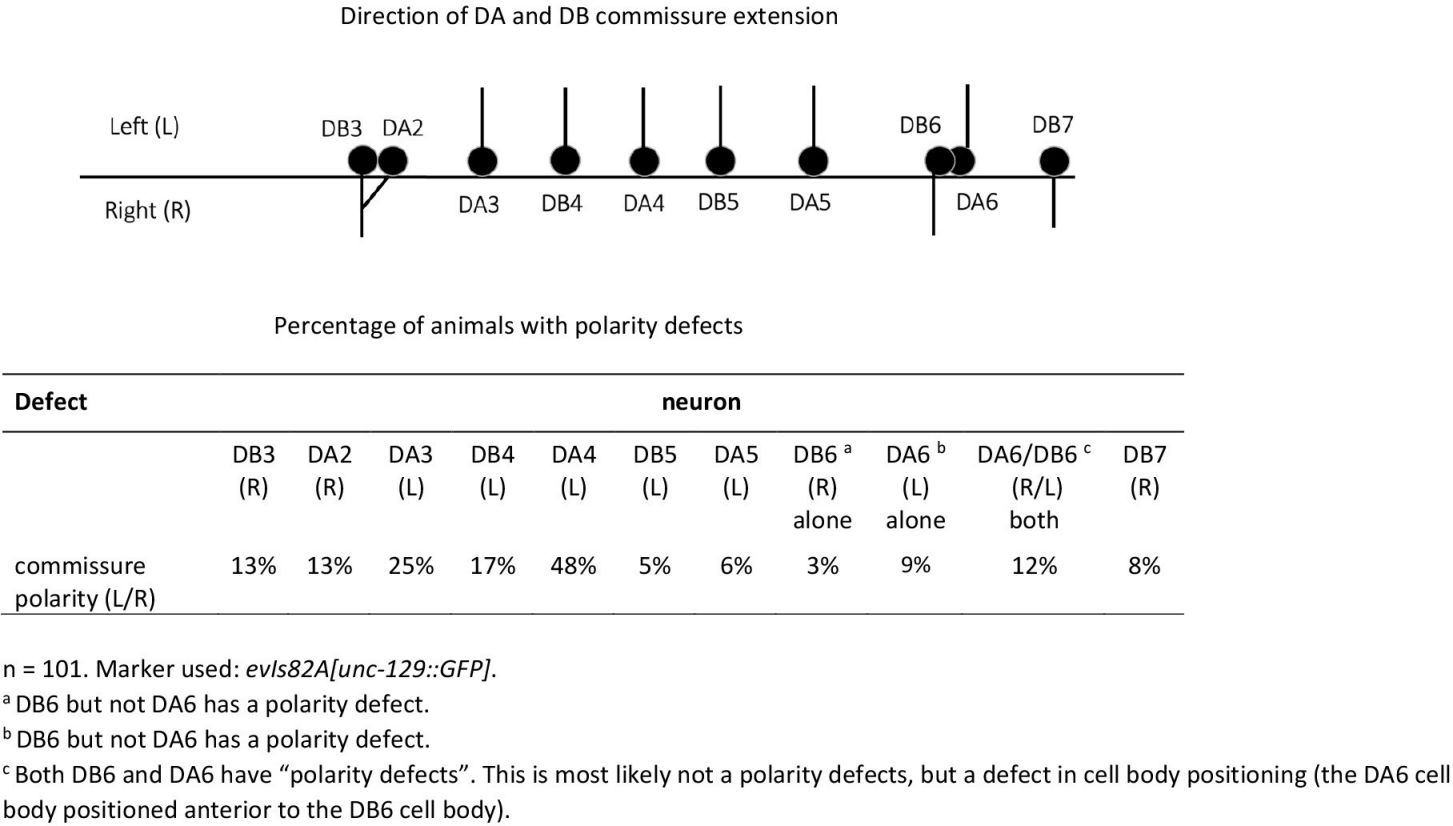
Polarity defects in individual DA/DB motoneuron commissures in *lron-11(ok2333)* mutants. The top part shown the direction of commissure extension (left or right) for individual DA and DB commissures. The table shows the percentage of animals, where commissures grow out on the wrong side, suggesting a left/right polarity defect of the neuron.

^a^ animals, where both DB6 and DA6 show commissure polarity defects were not counted as defective, since the underlying defect is most likely a cell body positioning defect (see also Fig 5)

Motoneurons innervating dorsal muscle cells send commissures from the ventral side of the animal to the dorsal side. The direction of commissure outgrowth or polarity is invariant for each individual motoneuron. Almost all DD/VD commissures extend up the right side of the animal (Fig 4A). Conversely, some DA/DB motoneurons send commissures up the left side. For example, DB3 and DA2 commissures always extend up the right side, whereas DA3 and DB4 commissures invariably extend up the left side (Fig 4F). We found that DD/VD commissures showed a moderate defect in the polarity of commissure navigation with 55% of animals having at least one commissure extend up the wrong side of the animal (the marker strain had 34% penetrance for commissure polarity defects) (Table 3 and Fig 4C). DA/DB commissures show much more severe polarity defects with 76% of the *lron-11(ok2333)* mutant animals having at least one commissure with a polarity defect (Table 3 and Fig 4G). The majority of animals had one or two commissures extending on the wrong side, and a small percentage (7%) had 4 or more commissures with polarity defects (Table 3). When we evaluated individual DA/DB commissures, we found that some neurons showed a high penetrance of defects, whereas others showed almost no defects (Fig 5). The highest penetrance of polarity defects (48%) is found in the DA4 motoneuron, whereas DB5, DB6 and DA5 motoneurons only show 4–6% defects. For DB6 and DA6 commissures we noticed that the coincident occurrence of both DB6 and DA6 commissures showing polarity defects was very high given the percentage of animals with only DB6 or DA6 polarity defects. Since the DB6 and DA6 cell bodies are very close to each other it seems likely that the relative position of the DB6 and DA6 cell bodies was swapped in some mutant animals, leading us to erroneously conclude that both commissures had polarity defects in that animal (Fig 5). There is no obvious correlation between the polarity of outgrowth (left versus right) and the severity of the defects. We did find significant gaps in the DNC in *lron-11* mutants with the DD/VD specific marker (Fig 4E). DD/VD commissures should form a continuous tract in the DNC (Fig 4D), however in 23% of *lron-11* mutants there was at least one gap (Table 3). Perhaps commissure polarity defects can cause a commissure to enter the DNC at a different point, a subtle navigational defect which could result in gaps in the DNC. Or *lron-11* might play a minor role in the DNC neurite guidance.

When observed on a culture plate, *lron-11(ok2333)* mutant animals move normally and do not show obvious signs of uncoordinated movements. They respond to touch as expected, by backing away. Animals look normal in their overall appearance and do not show obvious morphological defects. Taken together this suggests that there are no major defects in either muscle or hypodermal development.

## Discussion

This study focuses on the *C*. *elegans lron* gene family, which have not been previously studied in any context, including axon guidance, with the exception of *dma-1* [22, 27, 28]. The *lron* gene family consists of 16 eLRR genes that contain only LRRs in their extracellular domain. These *lron* genes encode mostly single-pass transmembrane proteins, though some are GPI-linked or secreted. We systematically investigated the *lron* family to identify if any of these genes function in axon guidance in the *C*. *elegans*’ VNC. We found that *lron-11* is important for the guidance of axons of several different types of neurons.

### VNC pioneer navigation defects

The AVG neuron pioneers the right VNC axon tract and the PVPR neuron pioneers the left tract of the VNC. Only one of the *lron* mutants, *lron-11*(*ok2333*), showed weakly penetrant AVG navigation defects. However, since the second allele, *lron-11*(*gk5321*), did not show AVG defects, *lron-11* is unlikely to play a role in AVG navigation. We found 23% PVPR navigation defects in *lron-5*(*gk959442*). However, in a second allele *lron-5*(*gk5278*), we did not see significant defects, suggesting that the defects in the *lron-5*(*gk959442*) strain are likely due to background mutations. In contrast, we found significant PVPR defects in both *lron-11* alleles, suggesting that *lron-11* is important for PVPR axon navigation.

Several pathways are known to be involved in PVPR axon guidance. The cadherins *cdh-4*, and *fmi-1*, as well as the *lin-17*/*frizzled* receptor display the largest PVPR defects, with a crossover penetrance of over 50% [29, 30]. Additionally, the ephrin pathway functions to inhibit several axons, including PVPR, from crossing the ventral midline [31]. The ephrin receptor is expressed in the extending growth cone, and ephrins and the IgCAM *wrk-1* signals from the surface of motoneuron cell bodies along the midline. *sax-3/robo* mutants display a high PVPR crossover penetrance as well (inferred from PVQL defects), however this appears to be largely independent of its usual ligand *slt-1*/*Slit* [31, 32]. Additionally, *unc-6/netrin*, which is secreted by ventral cells [33], also results in penetrant PVPR axon guidance defects when mutated [34]. Finally, there are two PVPR axon guidance pathways mediated by heparan sulfate modified proteins. Heparan sulfate modifying enzymes *hst-2* and *hse-5* mutants have ~50% PVPR crossover defects, though the protein they are modifying has not been identified [35]. *hst-6* may modify the HSPG *sdn-1*, which acts in a parallel PVPR axon guidance pathway [36].

With the data currently available, it is not possible to determine which of these pathways *lron-11* might be a part of, if any. The *lron-11* mutant’s PVPR crossover defects that we observed were more moderate than in most of these other known axon guidance genes. The exception is the ephrin *vab-2*, which also has PVPR defects under 20% [31]. Currently there is no evidence of LRR domains interacting with ephrins or their receptors; the RTK eLRR TrkB binds ephA, but the LRR domain was not required for this interaction [37]. However, there is evidence that the addition of heparan sulfate to neurexin is necessary for its proper binding with the eLRR-only proteins LRRTM1 and LRRTM2 to induce synaptogenesis [38–40]. Furthermore, heparan sulfate is also required for a functional interaction between LRRTM4 and glycipans, a heparan sulfate proteoglycan (HSPG) [41]. So, it is possible LRON proteins like LRON-11 bind HSPGs. Alternatively, Robo receptors have been shown to interact with several different eLRR proteins [16, 42–45]. This includes the eLRR protein SLT-1/Slit, where the ligand’s LRR domain is essential for this interaction [16, 42]. Therefore, it is possible that LRON-11 is another eLRR protein that binds SAX-3/Robo. To determine which pathway *lron-11* acts in, genetic interaction studies are required where double mutants with *lron-11* and a candidate gene, such as *sax-3*, are generated and assessed for axon guidance defects. If the penetrance of defects in the double mutants is not increased compared to the single mutants, then the genes are predicted to act in the same pathway. Conversely, if the double mutant’s defects are more severe than either single mutant, then the two genes are acting in separate pathways. Further experiments are required to determine how the genes interact.

While the axon guidance pathways that *lron-11* is acting in are currently unknown, research on other similar eLRRs is informative as to how *lron-11* might function. *lron-11* has a similar structure to *dma-1*, which functions as an adhesion receptor, so *lron-11* might also act cell autonomously as an adhesion receptor. If *lron-11* acts as adhesion receptor, it would likely mediate adhesion to cells, such as muscle cells or hypodermal cells, that are adjacent to the outgrowing axon. Since PVPR is the first axon growing in the left VNC axon tract, it cannot simply adhere to earlier outgrowing axons. Alternatively, *lron-11* might act as receptor for a guidance cue cell-autonomously or act as a guidance cue non cell-autonomously.

Identifying where *lron-11* is expressed would help determine in which cells *lron-11* is required for axon guidance. A detailed RNAseq dataset shows that *lron-11* is expressed primarily in neurons during embryogenesis, when axons are extending [21]. Expression was found in various motoneurons and interneurons including PVP (PVPL and PVPR data was combined in this dataset). Expression was also observed in hypodermal and muscle cells. This broad expression makes it difficult to determine the site of action for *lron-11*. Future experiments, such as rescue experiments, would be required to determine in which cells *lron-11* is required to guide the PVPR axon.

The previous discussion assumes that the axonal defects in PVPR observed in *lron-11* mutants are due to navigation defects during axon outgrowth. An alternate explanation of why the PVPR axon sometimes ends up in the right VNC axon tract in *lron-11* mutants is that *lron-11* might be important to maintain axon position after the initial outgrowth. The position of neural processes within the VNC needs to be actively maintained when the animal starts to move after hatching. Maintenance defects can lead to axons “flipping over” into the wrong axon tract and are indistinguishable from initial navigation defects in adult animals. Maintenance defects have been found after ablation of the PVT neuron [46] and in several mutants [46–49]. These maintenance defects seem to specifically affect a small set of axons, mainly AVK, PVP, PVQ, HSN and RMEV axons [46–49]. Motoneurons axons in the VNC are not affected by experimental manipulations causing maintenance defects [46]. To examine the possibility of maintenance defects in *lron-11* mutants further, neurite defects need to be evaluated in newly hatched L1 animals before the onset of movement and compared to defects in older animals.

### VNC follower axon navigation defects

Both *lron-11* alleles showed significant defects in the navigation of VNC axons when scored with a pan-neuronal marker. Using cell-type specific markers we found that the defects are mainly due to navigation defects in command interneurons and DD/VD motoneurons, as well as the previously described PVPR defects. Several other classes of neurons were not affected, suggesting *lron-11* is not required for axon navigation in general, but has a specific function in certain classes of neurons. The penetrance of command interneuron defects was not very high, suggesting a modest role for *lron-11* in guiding command interneurons in the VNC. Mutants in the ephrin pathway and the cadherin *fmi-1* also have crossover defects in command interneurons, with a higher penetrance than *lron-11* mutants [30, 31].

We observed DD/VD neurites extend aberrantly into the left tract in *lron-11*(*ok2333*) mutant animals. This defect often occurred due to a crossover event, but sometimes this defect would be caused by an outgrowth error when the neurite would outgrow directly into the left tract. DD/VD neurites crossing into the left tract could be caused by loss of adhesion that would normally bind the neurite to other neurites in the right tract. Loss of adhesion is thought to be the cause of the cadherins *cdh-4* and *fmi-1*’s DD/VD neurite crossover phenotype [29, 30]. However, since some of the neurites initially outgrew into the left tract, this suggests that *lron-11* plays a signaling role in DD/VD neurite guidance within the VNC, as this navigation defect cannot be easily explained as an adhesion defect. Several signaling pathways are known to guide DD/VD neurites in the VNC. Mutations in the axon guidance cue *unc-6* and the receptor *sax-3* also have DD/VD crossover defects [34]. In addition, *hst-2* and *hse-5* mutants were shown to have penetrant ‘VNC defasciculation’ defects in DD/VD motoneurons as well [35]. Additional genetic experiments are required to determine if *lron-11* functions in one of these signaling pathways for DD/VD neurite guidance in the VNC.

### Commissure defects

In addition to navigation defects in the VNC, we found polarity defects in various classes of motoneurons affecting the direction of extension (left versus right) of commissures traveling from the VNC to the DNC (Fig 4C and 4G and Table 3). However, within the DNC we did observe gaps in the DD/VD commissures in over 20% of *lron-11* mutants (Fig 4E and Table 3). It is unclear whether the commissure polarity defects observed in *lron-11* mutants could be causing the DNC to become disorganized, or if *lron-11* plays a minor role in the DNC.

How LRON-11 functions in commissure polarity defects is not understood. Commissure polarity is known to have a signaling component, since *unc-6* and *sax-3* mutants have commissure polarity defects [34]. Additionally, heparan sulfate also appears to be involved in this process, with *hst-2* and *hse-5* animals having ~25% penetrant defects [35]. However, there is some evidence that adhesion, or at least cell-cell contact, is also important for commissure polarity. The cadherin adhesion receptors *cdh-4* and *fmi-1* both have commissure polarity defects, with *cdh-4* having over 50% penetrance for commissure polarity defects in DD/VD motoneurons [29, 30]. Further evidence comes from AVG ablation experiments. When AVG is ablated early, its axon is not able to pioneer the right tract, resulting in the right axon tract becoming highly defasciculated [5]. Interestingly, AVG ablation also causes a high penetrance of commissure polarity defects, in both DD/VD and DA/DB motoneuron commissures [34]. Therefore, adhesion to AVG or other neighbouring axons in the right tract might be important for the establishment of left-right polarity in motoneurons. Thus, LRON-11 could act as an adhesion receptor like DMA-1, or in a signaling pathway to mediate commissure polarity.

For DA/DB commissures we found that the penetrance of polarity defects in the *lron-11* mutant varies greatly among individual neurons. The most heavily impacted was DA4, which was defective in ~50% of animals, which is what you would expect if the polarity was random. This suggests that without functional LRON-11 DA4 seems unable to polarize along the left-right axis. Conversely, the posterior-most neurons evaluated, DB5, DA5, DB6, DA6 and DB7, were only slightly affected, with commissure polarity defects of 10% or less in the mutant. This suggests that *lron-11* is required to various degrees in different DA/DB motoneurons. Interestingly, the other mutants that cause penetrant DA/DB commissure polarity defects, the heparan sulfate modifying enzymes, *sdn-1* and *sax-3*, also have divergent effects on different DA/DB commissures. In *hst-2*; *hst-6* double mutants, and in *sdn-1* mutants the DA2 and DB3 commissures almost completely lost their left-right polarity, but no other commissures where greatly impacted [50]. Conversely, *sax-3* mutants most heavily impacts DA3 commissure polarity [50]. Taken together, these results suggest that different DA/DB motoneurons rely on different axon guidance pathways to polarize along the left-right axis. DA2 and DB3 seems to rely on a heparan sulfate pathway, DA3 relies primarily on *sax-3* and DA4 relies on *lron-11*. Presumably the other DA/DB motoneurons rely primarily on other genes for determining commissure polarity, or utilize two of these commissure polarity pathways redundantly. Since the process of determining commissure polarity is so heterogeneous among the A and B-type motoneurons this suggests that even similar neurons might rely on distinct processes for the same commissure polarity decision.

### Potential functions of other *lron* genes

Of the *lron* genes we tested, only *lron-11* was found to function in guiding axons in the VNC. The other *lron* genes could still have a role in guiding axons of neurons that were not analysed in this study. *lron-3*, *lron-5*, *lron-7*, *lron-9*, *lron-10*, *lron-12*, *lron-13*, *lron-15* and *dma-1* are all expressed in various classes of neurons during embryonic development, especially in the head [21]. Therefore, these genes could be important for axon navigation in parts of the nervous system that were not examined here, such as the nerve ring. Alternatively, they may act in other developmental processes that eLRR proteins in mammals are known to function in, such as neurite target selection or synaptogenesis [12, 51, 52]. While studies have shown that *dma-1* acts as an adhesion receptor for dendrite guidance in the FLP and PVD sensory neurons, whether it has a similar role in other neurons is not known [22–24, 27].

*lron* genes are also expressed in non-neuronal tissues. *lron-8*, *lron-9*, *lron-12* and *lron-15* are expressed in hypodermis and/or muscle during embryonic development [21]. These genes might be required cell-autonomously for the development of these tissues. It is also possible that some of these genes act to guide axons in lateral axon tracts that are in contact with hypodermal and muscle cells expressing *lron*-genes. Finally, *lron-1*, *lron-2*, *lron-6*, *lron-8*, *lron-15* and *dma-1* are expressed in pharyngeal cells and *lron-7* is expressed in the intestine during embryogenesis suggesting that members of the *lron*-family might have development roles in many different tissues [21, 53, 54]. While diverse functions of eLRR genes in mammals have been discovered [11, 22, 39, 55–61], this gene family remains understudied [7]. A further investigation into the function of *lron* genes in *C*. *elegans* could identify novel roles of mammalian eLRR genes.

## Conclusion

We analysed the *lron* gene family for axon guidance defects within the VNC and discovered significant axon guidance defects with a pan-neuronal marker and pioneer marker in *lron-11* mutants. The primary defect was interneuron and motoneuron axons crossing into the opposite VNC tract. In addition, we found polarity defects in the direction of extension of motoneuron commissures. Based on the molecular nature of *lron-11* as a transmembrane protein with LRRs on its extracellular domain, we propose that *lron-11* acts as a receptor for guidance cues or as adhesion molecule (or both in different contexts) in several different neuron subtypes. In mammals, many eLRR genes have not been well characterized, partially due to the limitations of working with complex and expensive model organisms. The identification *lron-11* as novel axon guidance gene in *C*. *elegans* provides a potential function for similar mammalian eLRR genes during nervous system development. Furthermore, research to discover which axon guidance pathway(s) *lron-11* is functioning in could uncover potential binding partners for mammalian eLRR proteins.

## Supporting information

S1 Fig AVK defects in *lron-11* mutants. Panel A shows wildtype, B and C show *lron-11* mutants. The anterior side of the animals are towards the left of the image, the left side of the animals is towards the top of the image. Scale bars: 20μm. A-C: AVKL and AVKR visualized with the marker *hdIs54[flp-1*::*GFP]*. A) In wildtype animals AVK axons extend anteriorly into the nerve ring, before exiting the nerve ring and running posteriorly along the VNC. B) In *lron-11(ok2333)* mutant animals AVK axons occasionally prematurely left the nerve ring (arrowhead). C) In *lron-11(ok2333)* mutant animals, AVK axons that prematurely left the nerve ring (arrowhead) traveled posteriorly but did not enter the VNC (‘v’ marks the position of the vulva).
(TIF)

S1 Table List of *lron* alleles and strains. (PDF)

S2 Table Primer pairs used to genotype *lron*-mutants. (PDF)

S3 Table Sequencing primers used to identify point mutation. (PDF)
