# Supplementary figures and images for: lron-11 guides axons in the ventral nerve cord of Caenorhabditis elegans
Source: PLoS One. 2022 Nov 30;17(11):e0278258. doi: 10.1371/journal.pone.0278258 (PMC9710760; doi:10.1371/journal.pone.0278258)

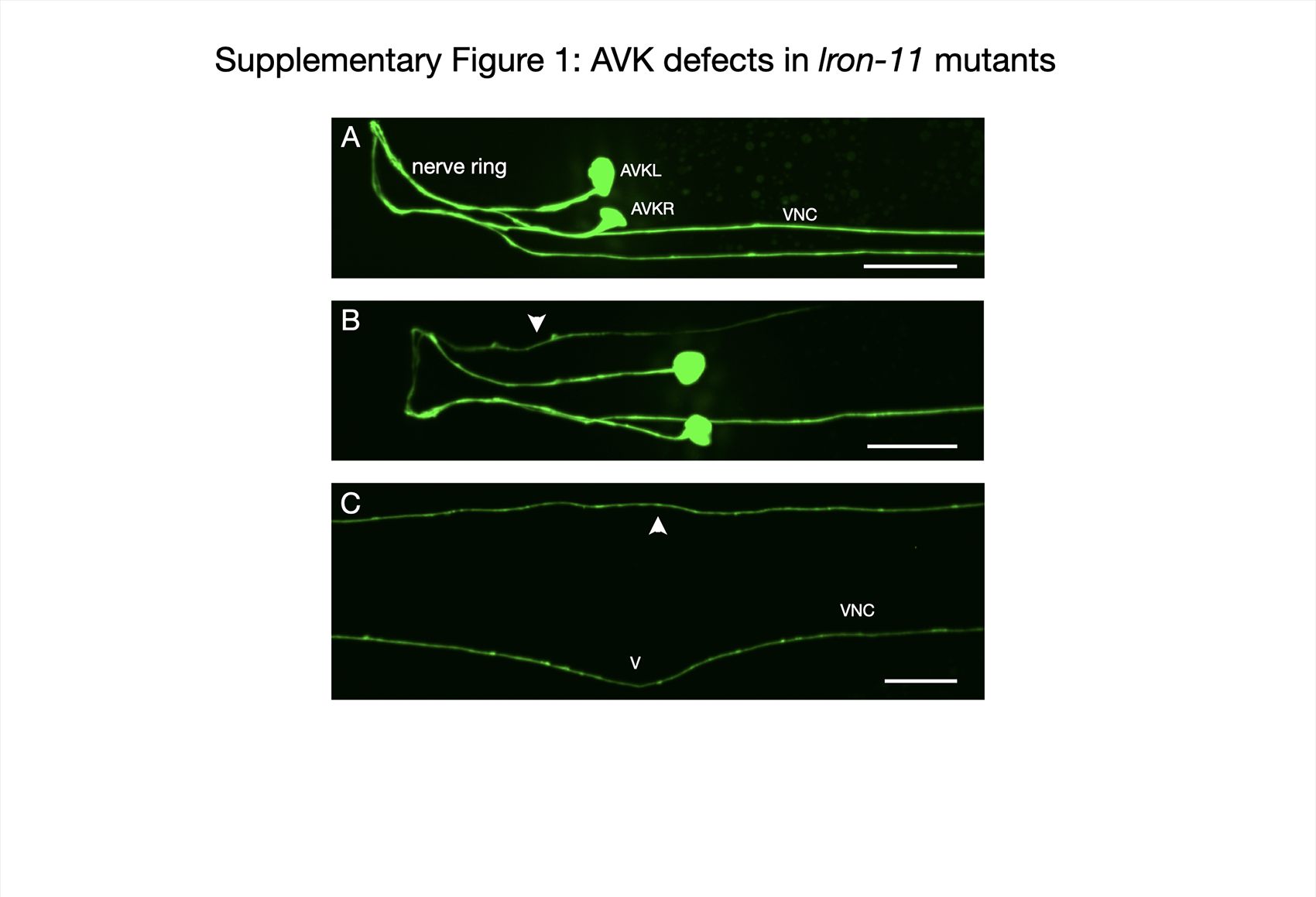

Supplement: S1 Fig — Panel A shows wildtype, B and C show lron-11 mutants. The anterior side of the animals are towards the left of the image, the left side of the animals is towards the top of the image. Scale bars: 20μm. A-C: AVKL and AVKR visualized with the marker hdIs54[flp-1::GFP]. A) In wildtype animals AVK axons extend anteriorly into the nerve ring, before exiting the nerve ring and running posteriorly along the VNC. B) In lron-11(ok2333) mutant animals AVK axons occasionally prematurely left the nerve ring (arrowhead). C) In lron-11(ok2333) mutant animals, AVK axons that prematurely left the nerve ring (arrowhead) traveled posteriorly but did not enter the VNC (‘v’ marks the position of the vulva). (TIF) [file pone.0278258.s001.tif]
