# Supplementary material for: lron-11 guides axons in the ventral nerve cord of Caenorhabditis elegans
Source: PLoS One. 2022 Nov 30;17(11):e0278258. doi: 10.1371/journal.pone.0278258 (PMC9710760; doi:10.1371/journal.pone.0278258)
Supplement: S1 Table — (PDF) [file pone.0278258.s002.pdf]

Supplementary Table 1: List of lron alleles and strains

Gene	chr	allele	strain	Mutation Type	source of mutation	outcrossed
lron-1	X	*gk5081*	VC4008	~3.5kb deletion of all exons + a ~5kb insertion[a]	CRISPR[b]	no
lron-3	X	*ok2614*	VH2941	~2kb deletion of exons 7-9, including the C terminus	mutagenesis[c]	4 times
	X	*gk5319*	VH2917	~4kb deletion of exons 5-8 and part of exon 9	CRISPR[b]	4 times
lron-4	II	*gk5099*	VC4026	~2kb deletion of exons 3-8 and part of exon 9	CRISPR[b]	no
lron-5	III	*gk959442*	VC41011	premature stop codon in exon 7, lacks its transmembrane domain	mutagenesis[c]	no
lron-5	III	*gk5278*	VC4295	~3kb deletion of part of exon 1, exons 2-7 and part of exon 8	CRISPR[b]	no
lron-6	I	*gk736335*	VC40637	premature stop codon in exon 10, lacks its transmembrane domain	mutagenesis[c]	no
lron-7	X	*gk5353*	VC4270	~2kb deletion of exons 4-10 and part of exons 3 and 11	CRISPR[b]	no
lron-8	I	*gk5317*	VC4231	~5kb deletion of all exons	CRISPR[b]	no
lron-10	III	*gk5064*	VC3992	~2kb deletion of part of the exon 1, exon 2 and part of exon 3	CRISPR[b]	no
lron-11	I	*gk5321*	VC4235	~ 3kb deletion of exon 3-5 and part of exon 6	CRISPR[b]	no
	I	*ok2333*	VH2839	~1kb deletion of most of the last exon, including the transmembrane domain	mutagenesis[c]	4 times
lron-12	III	*gk187625*	VC20146	premature stop codon in exon 5, lacks its transmembrane domain	mutagenesis[c]	no
lron-13	III	*gkDf31*	VC3229	~800bp deletion of exon 5, frameshift mutation [a]	mutagenesis[c]	no
lron-14	IV	*gk5340*	VC4256	~2kb deletion of part of exon 10, exons 11-14 and most of exon 15, lacks its transmembrane domain	CRISPR[b]	no
lron-14	IV	*gk401715*	VC20783	premature stop codon in exon 6, lacks its transmembrane domain	mutagenesis[c]	no

Gene	chr	allele	strain	Mutation Type	source of mutation	outcrossed
lron-15	II	*gk918201*	VC40994	premature stop codon in exon 5	mutagenesis[c]	no
dma-1	I	*wy686*	TV1624	~5kb deletion of all exons	*Mos1*-mediated deletion [b]	no

[a] This mutation was larger than predicted at Wormbase.org, which states that *gkDf31* is a 466bp deletion.
[b] Mutant strains generated with CRISPR/Cas9 or Mos1 do not contain background mutations. Therefore, these strains were typically not outcrossed. All CRISPR-generated strains contain a ~5kb insertion with a myo-2::GFP marker, which is expressed in the pharynx and allows for the easy identification of the presence of this allele in an animal.
[c] Mutant strains generated by chemical or radiation mutagenesis contain many background mutations; however these strains were outcrossed only, if the data suggested that the strain had axon guidance defects (see Table 1).
