# Supplementary material for: lron-11 guides axons in the ventral nerve cord of Caenorhabditis elegans
Source: PLoS One. 2022 Nov 30;17(11):e0278258. doi: 10.1371/journal.pone.0278258 (PMC9710760; doi:10.1371/journal.pone.0278258)
Supplement: S2 Table — (PDF) [file pone.0278258.s003.pdf]

Supplementary Table 2: Primer pairs used to genotype lron-mutants

Gene (allele)	upstream primer	downstream primer	wildtype Size (bp)	mutant Size (bp)
lron-1 **(*gk5081*)**	lron-1_gk5081_ex1 (TTTCTGGGACTTGACATACC)	lron-1_gk5081_P2 (TGGTGTATTGCTGATGGTTA)	655	0
lron-1 **(*gk5081*)**	lron-1_gk5081_insertionF (TACGTAGAGCTCGGTACCTC)	lron-1_gk5081_insertionR (GGTCGATTATCACTTTAGCA)	0	185
lron-3 **(*ok2614*)**	lron-3_ok2614_ex1 (CCCACATTTCTCATTCACTC)	lron-3_ok2614_ex2 (ATATTAAACCAAGACCCAACC)	2267	507
lron-3 **(*ok2614*)**	lron-3_ok2614_ex2 (ATATTAAACCAAGACCCAACC)	lron-3_ok2614_P1 (TAGGACAGGTGGATTTAGAAC)	1500	0
lron-3 **(*gk5319*)**	lron-3_ok2614_ex1 (CCCACATTTCTCATTCACTC)	lron-3_ok2614_P2 (GTGAAAGGTGGTAGATCAAAG)	1413	0
lron-3 **(*gk5319*)**	lron-3_ok2614_ex2 (ATATTAAACCAAGACCCAACC)	lron-3_ok2614_P1 (TAGGACAGGTGGATTTAGAAC)	1500	0
lron-4 **(*gk5099*)**	lron-4_gk5099_P1 (AGTTGGTATGAACCTTGGTG)	lron-4_gk5099_P2 (GGCTTACACATCAAACCAGT)	192	0
lron-4 **(*gk5099*)**	lron-4_gk5099_insertionF (TACGTAGAGCTCGGTACCTC)	lron-4_gk5099_insertionR (GGTCGATTATCACTTTAGCA)	185	0
lron-5 **(*gk959442*)**	lron-5_gk959442_geno1 (GAGGTTAGTCGGGAAGAACT)	lron-5_gk959442_geno2 (TCGATCCGCATACTTTCTAT)	509	509 (point mutation)
lron-6 **(*gk736335*)**	lron-6_gk736335l6geno1 (GAAGATAAAGAAGAACGAAGACG)	lron-6_gk736335l6geno2 (AGTGATGGCTATGTTGAGTG)	900	900 (point mutation)
lron-7 **(*gk5353*)**	lron-7_gk5353_ex3 (TTTGTGTGTCGTTTGTGTCT)	lron-7_gk5353_in4 (CACTTTAGCGATTACCAACC)	0	1598
lron-8(gk5317)	lron-8_gk5317_ex3 (TTCGGAGGAAGTGCTGAAGT)	lron-8_gk5317_ex4 (GAGGACCTTGCTGACGAC TC)	2027	0
lron-8(gk5317)	lron-8_gk5317_ex4 (GAGGACCTTGCTGACGACTC)	lron-8_gk5317_in3 (TGAATGAACTGCAGGACG AG).	0	2722
lron-10 **(*gk5064*)**	lron-10_gk5064_ex1 (CCACTCTTTGGGTCTCTGT)	lron-10_gk5064_P2 (GCGTCGACATAACTTCGT)	0	116
lron-10 **(*gk5064*)**	lron-10_gk5064_ex1 (CCACTCTTTGGGTCTCTGT)	lron-10_gk5064_ex2 (AGAGAGTTTCGGCTTCAAGA)	349	0
lron-11 **(*ok2333*)**	lron-11_ok2333_ex7 (TGACAGCGTACATCTTGG)	lron-11_ok2333_ex8 (TGACGGGAAAGAGGAAAGG)	1650	500
lron-11 **(*ok2333*)**	lron-11_ok2333_p5 (GTGCTCTTCATCCTTCTCCTG)	lron-11_ok2333_ex8 (TGACGGGAAAGAGGAAAGG)	450	0
lron-11 **(*gk5321*)**	lron-11_ok2333_ex7 (TGACAGCGTACATCTTGG)	lron-11_ok2333_ex8 (TGACGGGAAAGAGGAAAGG)	1650	0
lron-11 **(*gk5321*)**	lron-11_gk5321-in1 (TTCGGGGTGTAAAGTTCAGC)	lron-11_ok2333_ex8 (TGACGGGAAAGAGGAAAGG)	0	615

Gene (allele)	upstream primer	downstream primer	wildtype Size (bp)	mutant Size (bp)
lron-12 (gk187625)	lron-12_gk187625ex3 (CATCTAAAGAATTGGGCCTG)	lron-12_gk187625ex4 (AAAGAAGAGATTGATGGAAGATG)	1872	1872 (point mutation)
lron-13 (gkDf31)	lron-13_gkdf31_ex2 (CGGAGCGAAACTAGCAAT)	lron-13_gkdf31_P1 (GGAACAGTAAGTAAACGGTACG)	498	0
lron-13 (gkDf31)	lron-13_gkDf31_ex4 (TGACTTCCAGAAAATGCTTC)	lron-13_gkDf31_Ex5 (CAACAAAAACGACATTTCGAC)	1400	600
lron-14 (gk401715)	lron-14_gk401715_geno1 (TCCTGAAAACCTGACCGACT)	lron-14_gk401715_geno2 (TGAGCTAACGTGAGCAGCAT)	576	576 (point mutation)
lron-15 (gk918201)	lron-15_gk441339_geno1 (GAAACAACAAAGTTCGAAGG)	lron-15_gk441339_geno2 (TTTCGGAAGATCAGCTAGAG)	836	836 (point mutation)
dma-1 (wy686)	dma-1_wy686_ex1 (TCTATTTCCCACCCAACTGC)	dma-1_wy686_ex2 (ACACCGATCCGTCATTTTTC)	7000	3500
dma-1 (wy686)	dma-1_wy686_p1 (TCCTTTTTGCCGCACTACTT)	dma-1_wy686_ex2 (ACACCGATCCGTCATTTTTC)	985	0

Supplementary Table 3: Sequencing primers used to identify point mutations

Gene (allele)	Sequencing Primer (Sequence 5' to 3')	Wildtype Flanking sequence (5' to 3')	Mutant Flanking Sequence (5' to 3')	Point Mutation
Iron-5 **(*gk959442*)**	Iron-5_gk959442_seq1 (GAGGTATTAGTGGGACACGA)	TTTACGAGTTGATCAAAATCCTCTCCGATGTGATTGTTCCCTGTATGACAT	TTTACGAGTTGATCAAAATCCTCTCTGATGTGATTGTTCCCTGTATGACAT	C to T
Iron-6 **(*gk736335*)**	Iron-6_gk736335l6sequ2 (CAACTTCTCCACTCAACAATG)	GGAGCAATATGACTGGATGTTGGAACAAATGGAAGTTTATAGAGAATTAGA	GGAGCAATATGACTGGATGTTGGAATAAATGGAAGTTTATAGAGAATTAGA	C to T
Iron-12 **(*gk187625*)**	Iron-12_gk187625seq3 (GAAATACAATCGGAGACTTGG)	GATGAAATGGATGACTAGTGTTGAGGTAAGGATTTTATATGATTAAAAACC	GATGAAATGGATGACTAGTGTTGAGATAAGGATTTTATATGATTAAAAACC	G to A
Iron-14 **(*gk401715)***	Iron-14_gk401715_seq1 (GCAGAAGAATCCATTAACCA)	TGAAGTCATCAAGTTTTGCTGGTTCCAAATTTCAAGTCAAATTGTTTTTATCCGAGAATCCACTAC	TGAAGTCATCAAGTTTTGCTGGTTCCAAATTTTAAGTCAAATTGTTTTTATCCGAGAATCCACTAC	C to T
Iron-15 **(*gk918201)***	Iron-15_gk441339_seq3	AACACCAGAAGCTTTAAGAGATTTGCGAAATTTGACACATTTGAATCTAAA	AACACCAGAAGCTTTAAGAGATTTGTGAAATTTGACACATTTGAATCTAAA	C to T
