# Supplementary material for: lron-11 guides axons in the ventral nerve cord of Caenorhabditis elegans
Source: PLoS One. 2022 Nov 30;17(11):e0278258. doi: 10.1371/journal.pone.0278258 (PMC9710760; doi:10.1371/journal.pone.0278258)
Supplement: S3 Table — (PDF) [file pone.0278258.s004.pdf]

Supplementary Table 3: Sequencing primers used to identify point mutations

Gene (allele)	Sequencing Primer (Sequence 5' to 3')	Wildtype Flanking sequence (5' to 3')	Mutant Flanking Sequence (5' to 3')	Point Mutation
***Iron-5* (*gk959442*)**	Iron-5_gk959442_seq1 (GAGGTATTAGTGGGACACGA)	TTTACGAGTTGATCAAAATCCTCTCCGATGTGATTGTTCCCTGTATGACAT	TTTACGAGTTGATCAAAATCCTCTCTGATGTGATTGTTCCCTGTATGACAT	C to T
***Iron-6* (*gk736335*)**	Iron-6_gk736335l6sequ2 (CAACTTCTCCACTCAACAATG)	GGAGCAATATGACTGGATGTTGGAACAAATGGAAGTTTATAGAGAATTAGA	GGAGCAATATGACTGGATGTTGGAATAAATGGAAGTTTATAGAGAATTAGA	C to T
***Iron-12* (*gk187625*)**	Iron-12_gk187625seq3 (GAAATACAATCGGAGACTTGG)	GATGAAATGGATGACTAGTGTTGAGGTAAGGATTTTATATGATTAAAAACC	GATGAAATGGATGACTAGTGTTGAGATAAGGATTTTATATGATTAAAAACC	G to A
***Iron-14* (*gk401715*)**	Iron-14_gk401715_seq1 (GCAGAAGAATCCATTAACCA)	TGAAGTCATCAAGTTTTGCTGGTTCCAAATTTCAAGTCAAATTGTTTTTATCCGAGAATCCACTAC	TGAAGTCATCAAGTTTTGCTGGTTCCAAATTTTAAGTCAAATTGTTTTTATCCGAGAATCCACTAC	C to T
***Iron-15* (*gk918201*)**	Iron-15_gk441339_seq3	AACACCAGAAGCTTTAAGAGATTTGCGAAATTTGACACATTTGAATCTAAA	AACACCAGAAGCTTTAAGAGATTTGTGAAATTTGACACATTTGAATCTAAA	C to T
